# The surface lipoproteins of gram-negative bacteria: Protectors and foragers in harsh environments

**DOI:** 10.1074/jbc.REV120.008745

**Published:** 2020-12-10

**Authors:** Gregory B. Cole, Thomas J. Bateman, Trevor F. Moraes

**Affiliations:** Department of Biochemistry, University of Toronto, Toronto, Ontario, Canada

**Keywords:** surface lipoprotein, protein translocation, immune evasion, vaccine development, complement system, nutritional immunity, CCP, complement control protein, FH, Factor H, FHbp, FH-binding protein, Lf, lactoferrin, Lol, localization of lipoprotein, NHBA, Neisserial heparin–binding protein, OM, outer membrane, OMP, outer membrane protein, Sec, secretory, SLP, surface lipoprotein, T2SS, type II secretion system, TbpA, transferrin-binding protein A, TbpB, transferrin-binding protein B, Tf, transferrin, TPR, tetratricopeptide repeat, Vn, vitronectin

## Abstract

Gram-negative pathogens are enveloped by an outer membrane that serves as a double-edged sword: On the one hand, it provides a layer of protection for the bacterium from environmental insults, including other bacteria and the host immune system. On the other hand, it restricts movement of vital nutrients into the cell and provides a plethora of antigens that can be detected by host immune systems. One strategy used to overcome these limitations is the decoration of the outer surface of gram-negative bacteria with proteins tethered to the outer membrane through a lipid anchor. These surface lipoproteins (SLPs) fulfill critical roles in immune evasion and nutrient acquisition, but as more bacterial genomes are sequenced, we are beginning to discover their prevalence and their different roles and mechanisms and importantly how we can exploit them as antimicrobial targets. This review will focus on representative SLPs that gram-negative bacteria use to overcome host innate immunity, specifically the areas of nutritional immunity and complement system evasion. We elaborate on the structures of some notable SLPs required for binding target molecules in hosts and how this information can be used alongside bioinformatics to understand mechanisms of binding and in the discovery of new SLPs. This information provides a foundation for the development of therapeutics and the design of vaccine antigens.

## Introduction to surface lipoproteins

Lipoproteins are soluble hydrophilic proteins that remain associated with a lipid bilayer through a covalently attached lipid anchor (1). It is well known that many lipoproteins are found in the periplasm and are tethered to the inner leaflet of the outer membrane (OM) or the outer leaflet of the inner membrane. Due to a previous underappreciation of lipoprotein export pathways, improvements in technology to detect and characterize surface proteins, and an interest in finding vaccine antigens, there have been a growing number of reports of lipoproteins coating the outer surface of gram-negative bacteria. These surface lipoproteins (SLPs) are structurally and functionally diverse and play critical roles in nutrient acquisition, immune evasion, cellular adhesion, and cell signaling (2). Although gram-positive organisms display SLPs as well, their synthesis and display vary from those of gram-negative organisms (3) and are beyond the scope of this review. In gram-negative organisms, the lion’s share of SLP-mediated immune evasion occurs by overcoming host nutritional immunity and the host complement system. Thus, the importance of SLPs to bacterial virulence and accessibility to host antibodies make SLPs ideal targets for vaccine design.

Nutritional immunity is the process by which hosts limit the availability of metals in circulation by expressing metal chelating proteins (*i.e.*, transferrin [Tf], lactoferrin [Lf], S100 proteins), ensuring that invading pathogens are starved for these critical nutrients (3). In a more aggressive defensive mechanism, the complement system provides the primary innate immune response, driving the formation of immune attractants and labeling invading pathogens for lysis and/or phagocytosis (4). Pathogen SLPs facilitate immune evasion from nutritional immunity or complement by binding the host proteins of these systems, reversing their roles and making them advantageous for the bacterium. To overcome nutritional immunity, SLPs bind directly to host metal sequestration proteins and facilitate the piracy of metal nutrients from the host. Likewise, to evade the complement system, SLPs bind host complement regulatory proteins whose presence on the surface inhibits the assembly of complement machinery and prevents damage to the bacteria.

In this review, we will provide an overview of our current understanding of immune evasion SLPs from gram-negative bacteria, reviewing their biosynthesis, molecular mechanisms, and viability as vaccine antigens. Pharma has already examined the value of SLPs as potential vaccine antigen endeavors that have culminated in the use of SLPs as antigens in Bexsero and Trumenba, Food and Drug Administration–approved vaccines from Glaxosmithkline and Pfizer, that have been developed to protect against serogroup B *Neisseria meningitidis* (5, 6). The development of new SLP-based vaccines to protect against other bacterial pathogens first requires initial SLP identification, through either bioinformatics or other biochemical techniques. Further understanding of SLP structure–function relationships, as we will discuss, can aid in the generation of mutants that can improve antigen effectiveness by optimizing stability or reducing target binding.

## The SLP journey: cytoplasm to surface display

### SLP biogenesis and processing

SLPs undergo a series of processing and trafficking events in order to reach the cell surface. These events have been intensely characterized in the model organisms, *Escherichia coli* and *Neisseria* species. Bacterial lipoproteins synthesized in the cytoplasm are kept in an unfolded, or translocation-competent, state by the chaperone secretory (Sec)B and delivered to the SecYEG channel for translocation across the inner membrane, which is driven by the SecA motor and relies on ATP hydrolysis (7). The nascent protein emerges from the ribosome with a Sec signal sequence that begins with a positively charged region followed by a hydrophobic stretch and a C-terminal polar region (7). Bacterial lipoproteins contain a consensus sequence in their N-terminal region called the lipobox motif, [LVI][ASTVI][GAS]C, which contains an invariant Cys residue that undergoes lipid modifications and becomes the first residue (+1 Cys) of the mature protein after the signal peptide is cleaved (8). While most lipoproteins including Neisserial SLPs go through the Sec translocon, lipoproteins with the TAT signal sequence, with the motif S/TRRXFLK, move across the inner membrane *via* the tat translocon (7, 9). The tat translocon relies on the proton motif force and can transport folded proteins (7).

**Figure 1:**
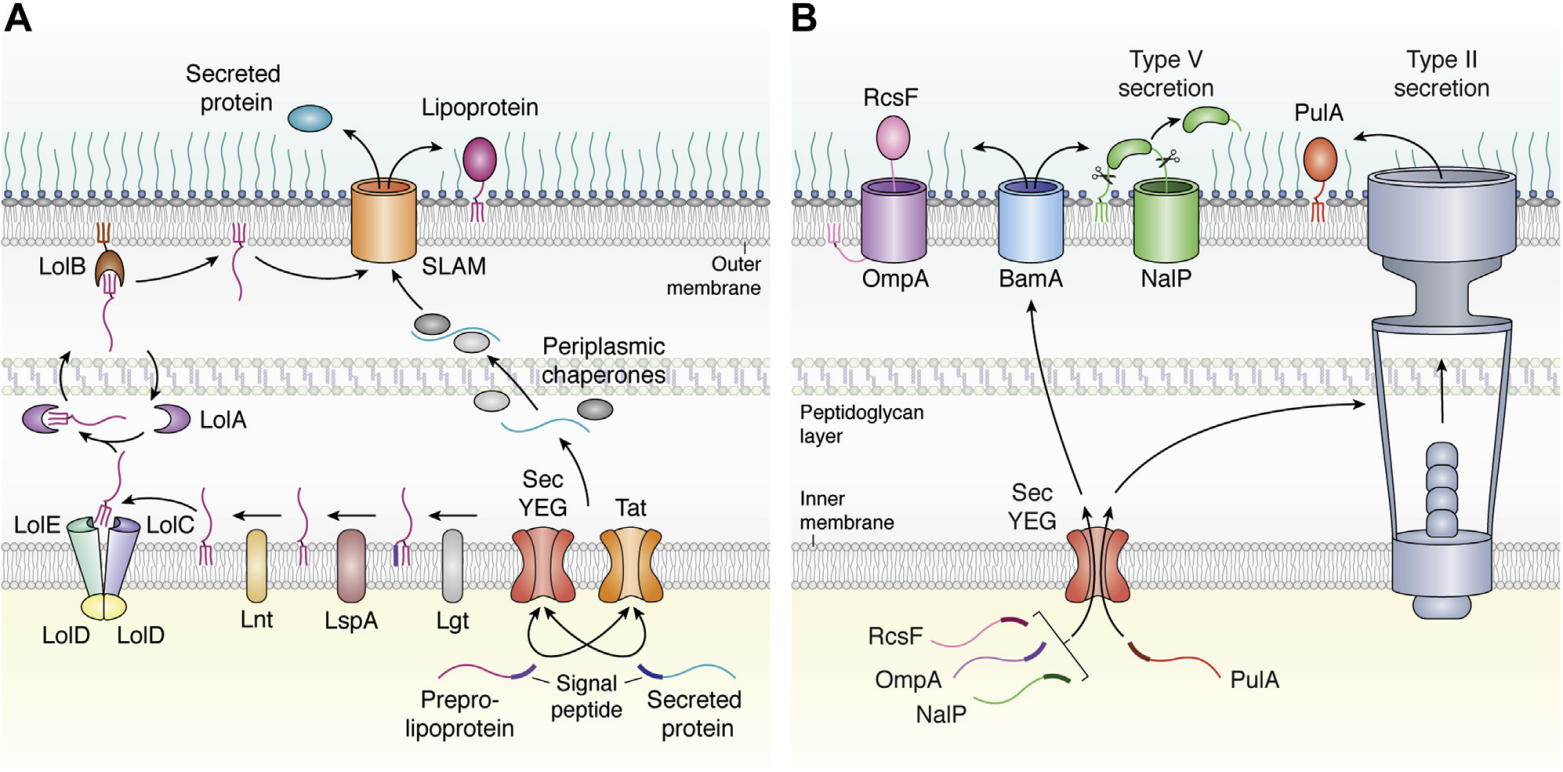
**Overview of lipoprotein trafficking to the outer membrane.***A*, preprolipoproteins are directed to the SecYEG or Tat translocon by a specific signal sequence for translocation across the inner membrane. Lgt, LspA, and Lnt are involved in lipoprotein precursor maturation, catalyzing removal of the signal peptide and addition of a lipid anchor. Lipoprotein trafficking to the outer membrane is facilitated by the Lol pathway, where a number of Lol proteins release the mature lipoprotein from the inner membrane (LolCDE), shuttle it across the periplasm (LolA), and facilitate insertion into the inner leaflet of the outer membrane (LolB). Slam is involved in the final step of translocating SLPs across the outer membrane and is hypothesized to secrete proteins containing a consensus secretion signal lacking a lipobox motif. These secreted substrates are likely delivered to Slam in a translocation competent state by periplasmic chaperones. *B*, additional protein machinery has been demonstrated to export lipoproteins to the bacterial cell surface. This includes the BAM complex, which mediates the insertion of outer membrane proteins such as OmpA in complex with the RcsF lipoprotein and NalP, a type V secretion system consisting of a C-terminal β-barrel and N-terminal lipoprotein passenger domain. The type II secretion system in *Klebsiella oxytoca* has also been shown to export the surface lipoprotein, PulA. SLP, surface lipoprotein.

Upon translocation across the inner membrane, preprolipoproteins undergo a series of post-translational modifications (Fig. 1*A*). First, the inner membrane protein diacylglycerol transferase (Lgt) catalyzes the transfer of diacylglyceryl from phosphatidylglycerol to the sulfhydryl group of the lipobox Cys (10, 11). The signal peptide of the intermediate prolipoprotein is cleaved by signal peptidase II (LspA), which relies on two catalytic aspartic acid residues and can be inhibited by the peptide antibiotic globomycin (12). Apolipoprotein N-acyltransferase (Lnt) catalyzes the transfer of an acyl chain from phosphatidylethanolamine onto the amino terminus of +1 Cys, which results in a triacylated protein (13).

The Sec-mediated translocation and subsequent processing of lipoproteins is largely conserved between gram-negative and gram-positive organisms (14). Additionally, Lgt and LspA are essential in proteobacteria, while Lnt is dispensable in some gram-negative species such as *N. gonorrhoeae*, which transport diacylated lipoproteins to the OM (15). The absence of Lnt is likely tolerated in these organisms owing to differences in the downstream ABC transporter machinery, which may have altered specificity for lipid modifications (15).

### Localization to the OM

SLPs anchored in the inner membrane must be released and trafficked to the OM in order to perform their cell surface functions (Fig. 1*A*). The localization of lipoprotein (Lol) pathway has been well studied in *E. coli* and is also involved in SLP trafficking in *N. meningitidis* (16). LolCDE is an ABC transporter responsible for the release of lipoproteins from the inner membrane (17). Release is dependent on ATP hydrolysis and LolA, which shuttles the SLP to LolB, an OM lipoprotein that facilitates insertion of the SLP into the inner leaflet of the OM through an unknown mechanism (17, 18, 19). LolA docks onto LolC and receives lipoproteins from LolE (20, 21). Although the structure of LolCDE alone or in complex with a lipoprotein substrate has yet to be solved, the structures of LolA and LolB have shed insights into the mechanism of transport. LolA and LolB form incomplete β-barrel structures with alpha helical lids forming a hydrophobic cavity for encapsulating the acyl chains of lipoproteins for transport through the aqueous periplasm. Site-specific photo-cross-linking revealed that LolA and LolB interact in a mouth-to-mouth manner, where their hydrophobic cavities meet facilitating lipoprotein transfer to LolB (22). To ensure unidirectional transfer, LolB has a higher affinity for lipoprotein acyl chains and LolA contains a 3_10_ helix to prevent nonspecific localization to membranes and therefore retrograde transfer to the inner membrane (22, 23).

Studies have demonstrated that amino acids adjacent to +1 Cys dictate whether a lipoprotein is retained in the inner membrane or released by the LolCDE complex. In *E. coli*, aspartic acid at position +2 causes retention of lipoproteins in the inner membrane (24). In *Pseudomonas aeruginosa*, lysine at position +3 and serine at position +4 are important for inner membrane localization. However, the existence of specific sorting signals dictating interactions with the lol machinery is questionable as recent work has shown that the LolCDE machinery from *E. coli* can correctly sort lipoproteins in a LolCDE-deficient strain of *P. aeruginosa* (25). It has been proposed that the amino acid composition at the N terminus of lipoproteins determines the affinity for inner membrane phospholipids and thus retention (25).

Examining the prevalence of the Lol protein machinery in bacteria raises questions whether additional factors are important in SLP trafficking. LolA and LolCDE are highly conserved in gram-negative bacteria, and LolB is found only in β- and γ-proteobacteria (2). *N. gonorrhoeae* lacks LolC and LolE but instead contains LolF which appears to be a LolC and LolE hybrid, and this may account for the ability of this Lol system to transport diacyclated SLPs (15). While LolCDE is essential, LolA and LolB deletions are tolerable. Deletion of LolA and LolB causes the toxic buildup of lipoproteins like lpp in the inner membrane which can form cross-links with peptidoglycan and cause cell lysis (26). Deletion of these toxic lipoproteins results in viable cells that correctly traffic OM lipoproteins such as BamD, which suggests an alternative Lol-independent pathway downstream of LolCDE for lipoprotein localization exists (26). The ability of SLPs to traverse the periplasm to the inner leaflet of the OM also requires passage through the peptidoglycan, a mechanism that has yet to be completely elucidated.

### SLP delivery to the cell surface

Different strategies exist for delivery of lipoproteins across the OM to the cell surface. We provide a description of how SLPs are transported to the surface *via* the newly discovered Slam outer membrane protein (OMP) (discussed below) and a brief overview of other systems that have been shown to be involved in the translocation of specific lipoproteins.

The type II secretion system (T2SS) is a large apparatus thought to span both the inner membrane and OM (Fig. 1*B*) of many pathogenic and nonpathogenic gram-negative bacteria (27). A wide variety of folded proteins enter the T2SS from either the cytoplasm or the periplasm and are secreted through to the extracellular space by ATP hydrolysis. Among the substrates of the T2SS is the pullulanase lipoprotein from *Klebsiella oxytoca*, which functions as a starch-debranching enzyme (28).

Lipoproteins can also belong to the type V secretion system. Often called autotransporters, proteins of the type V secretion system contain a β-barrel translocation domain that facilitates transport of a secreted, so-called passenger domain across the OM (29). The Bam complex facilitates insertion of the autotransporter β-barrel into the OM. The inserted autotransporter then acts as a channel through which the passenger domain can translocate through to the cell surface (Fig. 1*B*) (30). The passenger domain is kept in an unfolded state by chaperones, and it is thought that the folding of this domain provides the energy for translocation (31). The Neisserial protein NalP is a lipoprotein consisting of an N-terminal β-barrel transport domain and a C-terminal passenger domain (30). After transport across the OM, folded NalP cleaves itself from its autotransport domain but remains associated with the cell surface (*via* its lipid anchor) where it functions in processing other Neisserial surface proteins (32).

The Bam complex is responsible for the insertion of β-barrel membrane proteins into the OM. In a recent report from enterobacteria, the lipoprotein RcsF was found to be translocated by the conformation cycling of BamA (Fig. 1*B*) (33). In this model, a folded RcsF binds to the inner lumen of BamA and an incoming OMP substrate triggers an inward-to-outward transition in BamA, resulting in translocation of both proteins into an OMP–RcsF complex (33, 34).

Many Neisserial SLPs rely on an OMP called surface lipoprotein assembly modulator, Slam, for transit across the OM and surface display (16). Slam was first discovered in *N. meningitidis* using a transposon mutagenesis screen to find genes important for transferrin-binding protein B (TbpB) surface localization and is required for Neisserial virulence (16). Slam consists of an N-terminal domain containing two tetratricopeptide repeats (TPRs) and a predicted C-terminal 14-stranded β-barrel (16). Two Slam homologs were identified in *N. meningitidis* that differ in their substrate specificity: Slam1 translocates TbpB and the Lf-binding protein (LbpB), while Slam2 is specific for HpuA (16). Although TbpB was shown to interact with Slam during translocation, it is still unclear whether Slam is a translocon or a chaperone that acts in concert with another OMP assembly complex such as Bam or Tam (35).

Slams are a family of OMPs present in a wide range of gram-negative bacteria (36). They are found predominantly in all clades of the proteobacteria, including human and animal pathogens, commensals, and bacteria found in the environment (36). Genes encoding Slam can be found adjacent to their substrate SLPs in many cases (36). Recent work found that there are different classes of Slams that can be clustered according to the lifestyle of the bacteria (animal pathogen, for example) and the type of substrate they are predicted to export such as lipidated SLPs or nonlipidated substrates (37). It is conceivable that Slams could play a role in secretion of proteins into the extracellular environment as TbpB with a mutated Cys residue can be found in the culture supernatant, suggesting that lipidation is not a strict requirement for translocation (38). A more recent example comes from the discovery of a novel secreted heme-scavenging protein called hemophilin produced by *Haemophilus haemolyticus* (39). Hemophilin is architecturally similar to the Neisserial SLPs, and we discovered that it is adjacent to a putative Slam-encoding gene, further implicating Slam in secretion. Most of our knowledge of Slams comes from one subcluster involved in translocation of lipidated Neisserial SLPs (37), highlighting the need to begin examining Slams from other classes. This may reveal new insights into Slam function and expand our knowledge of the repertoire of Slam-dependent substrates, an area of critical importance as Slams have been discovered in many pathogenic proteobacteria (36). Finally, the lack of Slam homologs in other phyla of bacteria that display SLPs suggests that additional translocation systems remain to be discovered.

## SLP roles in overcoming nutritional immunity

With the knowledge of SLP biogenesis in hand, we can begin to understand the functions SLPs perform in immune evasion and nutrient acquisition. Bacteria require transition metals such as Fe, Zn, Mn, Co, Ni, and Cu for growth and survival (3). These trace nutrients play critical structural and enzymatic roles in processes ranging from DNA replication to cell metabolism and respiration (3). Mammals sequester these metals, limiting their availability to invading bacteria as a method of growth restriction, a mechanism termed nutritional immunity (3). This term was initially applied to iron, the most abundant metal cofactor well studied for its role in bacterial pathogenesis. The definition of nutritional immunity has since been broadened to apply to other key nutrients.

Mammalian restriction of metals in the mucosa and blood is mediated by several proteins. Iron is stored within ferritin intracellularly, and any free ferric iron is tightly sequestered by the glycoproteins transferrin and Lf in sera and at mucosal surfaces, respectively (40). Coupled with its poor solubility at physiological pH, ferric iron exists at a concentration of 10^−18^ M in extracellular fluids, far below the 10^−7^ to 10^−5^ M requirement for optimal bacterial growth (41). Approximately 70% of iron in humans is found in heme, 95% of which is bound to proteins (42). Heme concentrations are under tight control as any hemoglobin (Hb) released from red blood cell lysis is bound by haptoglobin for clearance in the liver (42). Free heme released into the blood by Hb oxidation is also scavenged by the high-affinity heme-binding proteins, hemopexin and serum albumin (42).

Zinc is another important metal for enzymatic reactions and structural motifs, and manganese plays an important role in the bacterial response to counter oxidative stress caused by the immune system (43). Like iron, these trace nutrients are not freely available, with Zn levels reported to be in the picomolar range in blood (44). Restriction of Zn and Mn is achieved by the S100 proteins, a family of homo-dimeric EF-hand calcium-binding proteins (43). S100A8 and S100A9 form a heterodimer called calprotectin that binds Zn and Mn with high affinity, while S100A12 binds to zinc and copper. S100A7 also seems to have antimicrobial properties through chelation of zinc and is released by neutrophils at sites of infection (43).

Both obligate and opportunistic pathogens must overcome nutrient restriction to colonize and infect the host. Pathogens have evolved nutrient acquisition systems ranging in complexity from those based on simple diffusion (porins), as well as more elaborate protein machinery, including a family of SLPs that work in conjunction with membrane-imbedded TonB-dependent transporters (TBDTs), reviewed more thoroughly in (45). In this system, TBDTs bind and extract metals from host proteins, transporting them into the periplasm. SLPs function by extending beyond the membrane and lipopolysaccharide coating, thus being more accessible to their host protein targets. SLPs increase the local concentration of host metal carrier proteins at the surface and eventually hand off the target protein to a TBDT.

### Acquiring iron with TbpB and LbpB

**Figure 2:**
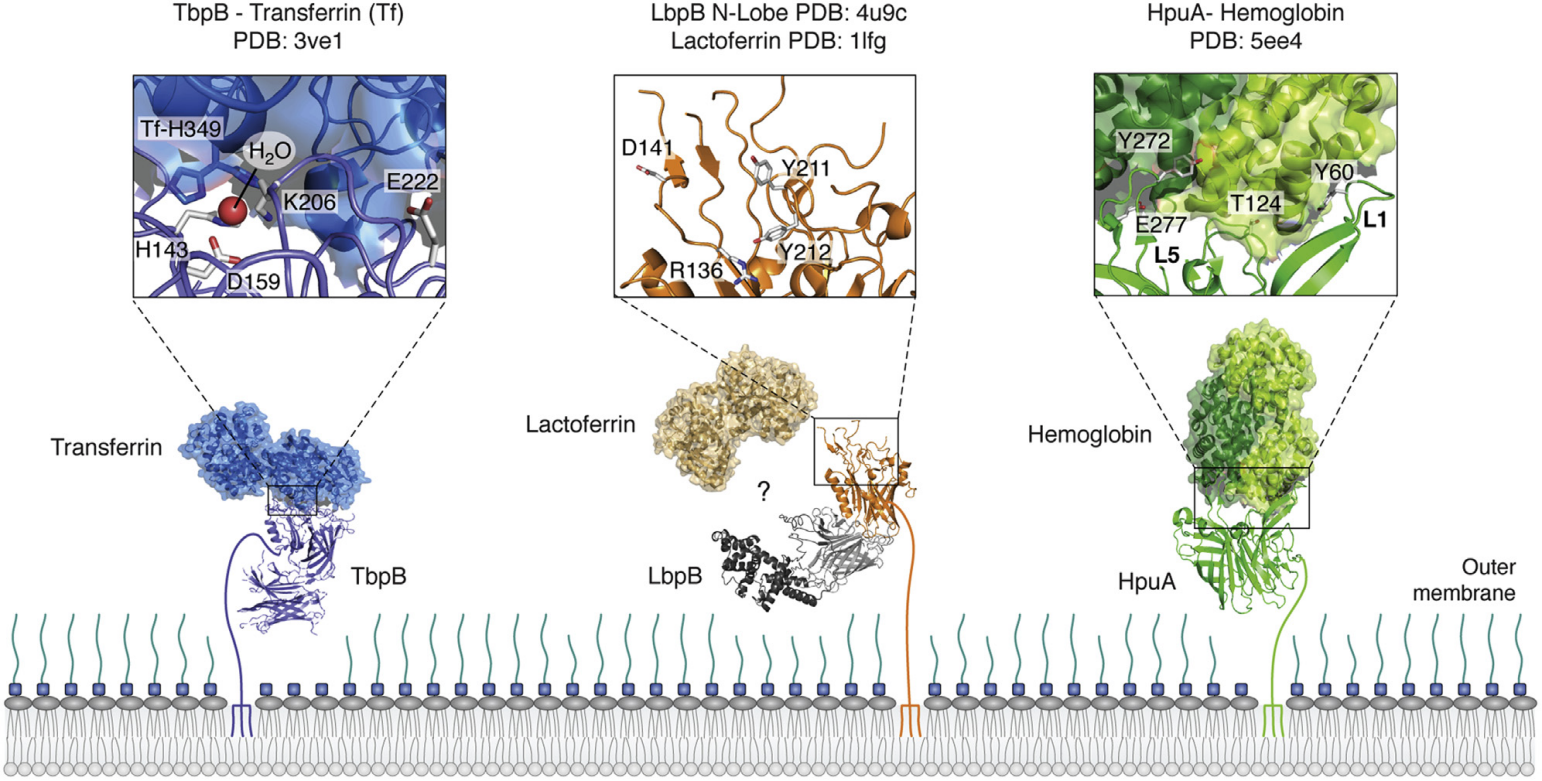
**Structures of Slam-dependent surface lipoproteins involved in overcoming nutritional immunity.** TbpB, LbpB, and HpuA are surface lipoproteins that work as bipartite receptors with their partner TonB-dependent receptors to capture and transport host-restricted nutrients across the outer membrane. Important residues involved in the binding interface are shown as stick representations and labeled. Individual structures of LbpB and its substrate lactoferrin are shown as the structure of the complex remains to be solved. The structure of the LbpB N-lobe has been solved, and a model of the C-lobe generated by I-Tasser is shown in gray scale. The C-lobe contains a highly charged helical region in *dark gray* that interacts with the lactoferrin N-lobe. HpuA loops 1 and 5 are labeled as these loops are important for binding β and α hemoglobin, respectively.

A plethora of mammalian pathogens bind to the iron transport protein transferrin (Tf) through an SLP defined as TbpB, or the transferrin-binding protein. These pathogens include *N. meningitidis/gonorrhoeae*, *Moraxella bovis*, and *Actinobacilus suis*, which infect humans, cows, and pigs, respectively. TbpB is a bilobed protein, made up of an N-terminal β-handle and an eight-stranded C-terminal β-barrel. The crystal structure of the TbpB–Tf complex (Fig. 2) shows that the N-lobe of TbpB binds and stabilizes the iron-loaded C-lobe of Tf (46, 47). The interface between the SLP and its target is large, burying a surface of ∼1450 Å^2^, and is made up primarily of hydrophobic interactions and a hydrogen bonding network; however, charge reversal mutants at the interface greatly reduced interaction affinity (46). It is presumed that once TbpB is bound to Tf, a hand-off of Tf to transferrin-binding protein A (TbpA) occurs, after which TbpA, a TBDT, extracts iron from Tf by wedging an α-helix into the iron-binding cleft of Tf, freeing iron for transport into the periplasm (47). TbpB binds only to iron-loaded Tf (hTf), whereas TbpA was shown to bind both apo and holo transferrin, suggesting that SLPs may also play a role in screening for only holo forms of target proteins (48). Size exclusion and electron microscopy analyses suggest that a ternary TbpB–Tf–TbpA complex may assemble (47). Binding studies using the TbpA/B system from porcine pathogens indicated that the lipid linker peptide is a requirement for ternary complex formation; however, it remains to be seen how critical ternary complex formation will be to proper function of the TbpA/B system (49, 50).

The sequestration of iron provides hosts with an innate defense, and subsequent bacterial iron piracy through TbpB has made transferrin the subject of pathogen-driven evolution (51). After many rounds of coevolution, the result is a pathogen adapted for infection of a single host species (52). An exception to this can be found in *Haemophilus somnus*, which has been shown to possess two different systems for the acquisition of iron from transferrins of multiple species (53). It has been recently proposed that two receptors *Hs*TbpA and *Hs*TbpA2 work in conjunction with an SLP, *Hs*TbpB, to broaden the transferrin recognition range to include transferrins from ovine, bovine, and caprine, allowing for *Histophilus somni* infection in these species (54).

Several Neisseriaceae and Moraxellaceae species have extended their iron piracy to include the use of Lf as an iron source. This glycoprotein has striking similarities to serum transferrin, including the ability to tightly but reversibly bind iron, as well as a high degree of structural and sequence similarity (55). As such, the bacterial mechanisms for high-jacking iron from Lf are also similar in nature; indeed, it is likely that Lf receptors were derived from transferrin receptors (56). Structures of the N-terminal lobe of SLP LbpB (Lf-binding protein B) show that it shares its core architecture with TbpB (Fig. 2). The LbpB N-terminal lobe is made up of an eight-stranded β-barrel and antiparallel β-strand handle domain (57, 58). Like TbpB and hTf, LbpB has been shown to favor binding to the iron-loaded form of hLf (59). Unlike TbpB, the C-terminal lobe of LbpB is made up of flexible anionic regions which have made structural determination of constructs with this domain difficult. However, this unstructured anionic domain may function to protect the bacteria from cationic antimicrobial peptides, suggesting the LbpB may play a role in multiple immune evasion strategies (60).

### Acquiring iron from heme with the HpuA–HpuB bipartite system

The Neisseriaceae family relies on a bipartite receptor composed of a surface lipoprotein (HpuA) and TonB-dependent transporter (HpuB) for heme acquisition (61, 62, 63). HpuA and HpuB are both required for growth on Hb and hemoglobin–haptoglobin (HbHp) as iron sources; however, HbHp is more supportive of growth (61, 64). Similar to other heme transporters, HpuAB does not strip heme of its iron and instead transports the intact heme molecule (65). Unlike transferrin acquisition by TbpAB, binding to hemoglobin is not a host-restricted phenomenon (66), nor is HpuAB selective of a heme-loaded substrate as shown by receptor binding to apo Hp (64).

Evidence for bipartite receptor formation comes from protease accessibility experiments in which the cleavage patterns of both receptors alone and together suggest complex formation (66). Binding experiments also showed robust Hb binding to cells in the presence of both HpuA and HpuB, with a reported k_d_ of 150 nM (66). The ability of HpuA and HpuB to form a coreceptor is advantageous as a flow cytometry–based cell assay demonstrated that HpuB binds to less hemoglobin on its own compared with cells expressing the HpuAB complex (64). Furthermore, HpuA expands the repertoire of substrates that HpuB can access as HpuB on its own binds weakly to HbHp (64).

The first glimpse into the molecular mechanism of Neisserial heme uptake came from the recent X-ray crystal structure of *Kingella denitrificans* HpuA bound to hemoglobin (Fig. 2) (67). In contrast to structures of bacterial heme scavengers, HpuA binds hemoglobin and not heme. HpuA consists of an N-terminal β-sandwich structure nested against a C-terminal 8-stranded β-barrel. HpuA binds to an α/β-hemoglobin dimer, on the face opposite to haptoglobin. Pull-downs confirmed HpuA binding to Hb and HbHp (67). HpuA contains two extended loops, L1 in the β-sandwich and L5 in the barrel that bind through various hydrophobic residues to the β- and α-subunits of Hb, respectively. Interaction with Hb is mediated through the globin chain and not heme, and no major conformational change occurs upon HpuA binding. Interestingly, L1 and L5 contain sites of positive selection, which may be explained by cycles of Hb escape and recapture as part of the molecular arms race between bacteria and their hosts (68). It remains to be elucidated if and how HpuA triggers heme release from Hb. The structure of the ternary complex (HpuAB–Hb/HbHp) is required for a complete picture of the molecular details of bipartite receptor formation, host hemoprotein binding, and heme transfer to HpuB.

There has been interest in using HpuA as a vaccine antigen because of its surface exposure and presence in the human pathogens, *N. meningitidis* and *N. gonorrhoeae*. Furthermore, HpuA is implicated in bacterial pathogenesis as 90% of disease-causing meningococcal isolates express HpuA along with another hemoglobin TonB-dependent transporter, HmbR (69). While HpuA is phase variable, it is present in the ON state in 90% of disease-causing isolates compared with 71% of carriage isolates (69). Unfortunately, the presence of HpuA in commensal Neisserial species and lack of bactericidal activity of polyclonal anti-HpuA sera raises concerns about the use of this SLP as an effective vaccine antigen (70, 71).

### Other nutritional bipartite systems

Recent structures of SLP bipartite systems in Bacteroidetes involved in oligopeptide and glycan uptake (72, 73) demonstrates that the field is expanding to include the study of SLPs that are involved in the uptake of nutrients that are not restricted by host-specific proteins. Bacteroidetes possess enzymes that degrade complex, inaccessible sugars and proteins to produce simpler molecules more amenable to uptake for growth and perhaps signaling (72, 73). X-ray and cryo-electron microscopy structures coupled with functional studies support a model in which the Bacteroidetes starch utilization system and receptor antigen gene SLPs function as a lid that operates according to a pedal bin mechanism. The SLP lid opens, binds its substrate, and closes for transport. In this model, the SLP is important for establishing the specificity of the bipartite receptor. Interestingly, Bacteroidetes do not contain Slams, and how these lipoproteins get to the cell surface is an area of future research.

## SLPs’ role in complement system evasion

SLPs also provide protection against other components of the immune system. The complement system is an ancient branch of the immune system that is responsible for the identification and removal of pathogens through a series of cascading enzymatic reactions. The early stages of the complement system can be divided into 3 pathways categorized by how target foreign bodies are recognized. In the classical pathway, the complement C1q complex recognizes antibodies (IgG and IgM) that have recognized specific antigens on an invader (74). In the lectin pathway, C1q is recruited by mannan-binding proteins that have recognized specific sugar patterns on bacterial surfaces (75). Finally, the alternative pathway is not dependent on a specific recognition factor but instead is initiated on random surfaces. The three pathways differ in how their respective enzymatic cascades are initialized, but all pathways converge in the formation of an enzyme complex called the C3 convertase, which drives the deposition of C3 (76). The surface buildup of C3 leads to phagocytosis of the opsonized body, or alternatively, the presence of C3 can drive the formation of the C5 convertase. The C5 convertase begins an enzyme cascade that results in the formation of the membrane attack complex which will lyse the invading cell (77).

The nature of the complement system requires that the host have a response to complement activation on host cells. It is crucial that the complement system is tightly regulated, especially in the case of the alternative pathway where complement is activated on random surfaces. Complement regulatory proteins come in two flavors, those that are membrane bound and expressed on the surface of host cells and those that are free-floating soluble proteins. Among the soluble group are C1-INH, Factor H (FH), the C4b-binding protein, and vitronectin (Vn) (78). Most examples of SLP-mediated complement evasion involve recruitment of a soluble regulator from the host, with FH binders being the most prevalent. We have chosen to highlight a diverse set of SLPs that function in complement evasion, the targets of which include heparin and the complement regulators FH and Vn.

### Neisserial heparin–binding antigen: an SLP for the general means of complement escape

**Figure 3:**
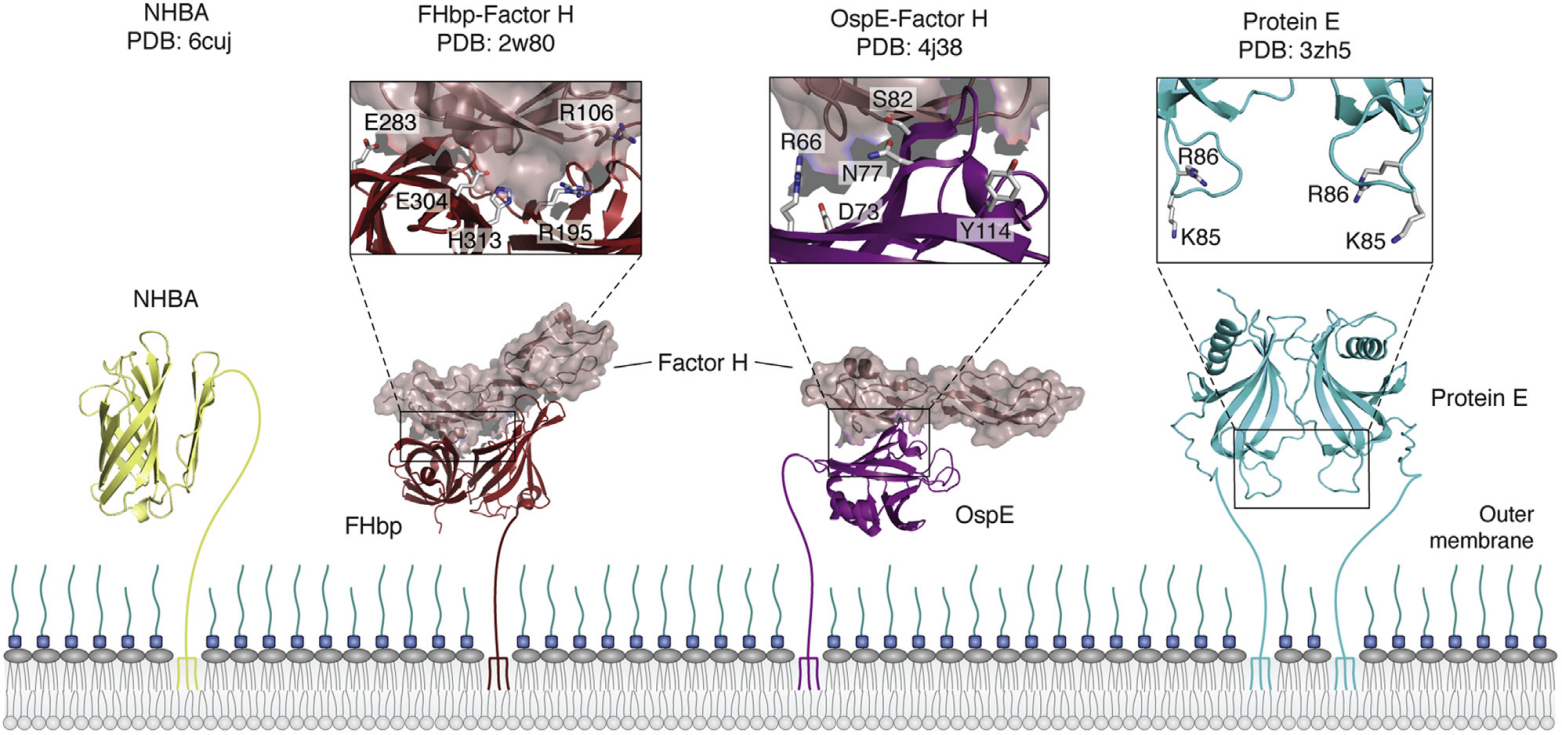
**Structures of surface lipoproteins involved in innate immune evasion.** NHBA and FHbp are *Neisserial* Slam-dependent surface lipoproteins involved in innate immune evasion achieved through the binding of heparin and Factor H, respectively. The NHBA binds to its ligand heparin through an arginine-rich region; however, it was not resolved in the available crystal structure. FHbp is shown bound to complement control protein domains 6 and 7 of human complement Factor H, and residues in the binding interface are shown as stick representations and labeled. OspE and protein E are surface lipoproteins not present in *Neisserial* species and are important for immune evasion. The mechanism of export is not known for these lipoproteins. Protein E residues K85 and R86 are critical for interaction with vitronectin, while residues R66, D73, N77, S82, and Y114 in OspE form the binding interface with Factor H. NHBA, *Neisserial* heparin–binding protein.

The Neisserial heparin–binding protein (NHBA) is responsible for capturing host heparin, a natural glycosaminoglycan. The NHBA is made up of a disordered N-terminal region followed by a 2-stranded β-hairpin and an 8-stranded C-terminal β-barrel domain (Fig. 3) (79). The structure of the C-terminal domain of the NHBA was first solved by solution NMR (80) and was later crystalized (81). The core structure of the β-barrel is identical in both structures, but there are differences in the observed conformations of the β-barrel loops, suggesting a degree of conformational flexibility here. Also, there are major structural differences in the positioning of the N-terminal β-hairpin, which was found to lie along one face of the β-barrel in the crystal structure, forming several hydrophobic contacts. In contrast, the β-hairpin from the NMR structure displayed a high degree of flexibility, which is thought to be the result of the presence of detergent in the NMR sample. As such, the crystal structure likely represents a more physiologically relevant structure. The β-barrel of the NHBA is structurally very similar to the barrels found in TbpB/LbpB and FH-binding protein (FHbp), but loops and the handle domain within these proteins contain key residues for binding their discrete substrates.

Neither of the NHBA structures to date contains the predicted disordered N-terminal domain, which contains the arginine-rich region responsible for heparin binding (80, 81). The mechanisms of why heparin binding is beneficial to the bacteria remain poorly understood, but binding does result in increased serum resistance in bacteria expressing functional NHBA. It is thought that the presence of heparin on the bacterial cell surface aids in evasion of the complement system as heparin has been shown to bind to several complement regulatory factors (82, 83). It is possible that heparin-mediated complement factor recruitment only functions to increase the surface concentration of regulatory factors so that they may be picked up by additional SLPs like FHbp, discussed below. Alternatively, heparin may form a polyanion shield around the cell surface, acting similar to the polysaccharide capsule present in some strains of *N. meningitidis* (84).

### FH-binding SLPs

The soluble complement regulator FH is a 150-kDa glycoprotein and major target of bacterial SLPs. Structurally, FH is made up of 20 complement control protein (CCP) regions that are sometimes called sequence consensus repeat regions. The presence of FH impedes the function of the C3 convertase either by accelerating the decay of the convertase or by acting as a cofactor for Factor I, an enzyme responsible for the decay of C3b into an inactive form. Both mechanisms effectively halt the cascade required for full complement activation (85, 86). Of the 20 CCP regions in FH, regions 1 to 4 are responsible for regulation of complement activity, and as such, bacteria do not bind to this area in order to preserve FH activity. There are two main binding sites that bacterial SLPs use to bind FH, CCPs 6 and 7, and CCPs 19 and 20, both of which contain glycosaminoglycan (*i.e.*, heparin)-binding sites that are critical to the host. Detailed experimental data on the role of Neisserial FHbp and how it binds FH CCPs 6 and 7 has provided insight into this interaction and how it could be exploited. CCPs 19 to 20 are bound by a wide range of pathogens including *P. aeruginosa*, *Haemophilus influenzae*, *Bordetella pertussis*, and others (78). However, we will limit our discussion to OspE from *Borrelia burgdorferi* as it has been crystalized in complex with FH domains (76, 87) providing structural and mechanistic details.

#### FHbp of *N. meningitidis*

The most well-characterized example of a bacterial hijacker of FH is *N. meningitidis* through its FHbp, an antigen which is used in both Trumenda and Bexsero vaccines (79). By binding FH to its surface, *N. meningitidis* is protected from complement-mediated cell killing (87, 88). The 27-kDa structure of FHbp is made up of two domains, both of which form contacts with FH, that are made up of β-strand and helical elements (87). The N-terminal barrel is made up of 6 antiparallel β-strands facing 2 shorter strands and a short α-helix. This is followed by an eight-stranded β-barrel and a short 3_10_ helix. The FH CCP regions 6 and 7 are bound to FHbp through extensive contacts (Fig. 3), burying a surface area of over 2800 Å^2^ (87). This is facilitated primarily through interactions between CCP and both barrel domains of FHbp, although there are also minor contacts to CCP 7 (87). The FHbp of circulating *N. meningitidis* strains can be divided into three variant groups (V1, V2, and V3), all of which are capable of binding FH with nanomolar affinity (89). Interestingly, each variant displays different residues that are critical for binding FH (89). Despite the extensive interface between FHbp and FH, there are several key residues that can modulate binding affinity in V1 variants. These residues are mostly polar in character. Point mutations of R195 and H313 showed at least a 5-fold reduction in FH affinity (90), while a double mutant E283A/E304A showed a 10-fold reduction in k_d_ (87). A single FHbp mutation, R106S, was able to abolish FH binding, and use of this mutant in vaccinations in mice resulted in an enhanced protective antibody response (91). Clearly mutational analysis can be used to fine-tune the immunogenic properties of an SLP antigen, but care must be taken as the FHbp double mutant, E283A/E304A, showed reduced serum bactericidal activity when compared with vaccinations using wild-type protein (92).

#### OspE from *B. burgdorferi*

The *Borrelia* genus is an extreme example of an SLP producer having over 80 predicted SLPs (93). The Borrelial SLP OspA was used in a vaccine that protected humans against Lyme disease. This vaccine was pulled from the market owing to side effects that were not detected in phase III clinical trials, and there is currently no available vaccine (94). *Borrelia* along with *Bacteroidetes* was found to produce SLPs, but no Slam homolog was found, a notable exception suggesting that there may be novel translocation mechanisms or novel Slams that remain to be discovered (36). It remains to be seen if any one Borrelial SLP is the most critical as each is likely important to different conditions based on tick feeding patterns. We chose to review OspE (outer surface protein E) as it was one of the few examples where the SLP–ligand complex structure has been solved.

OspE is a 17-kDa protein that begins with a classic unstructured region followed by a repeating structure of 4 β-strands followed by an α-helix (-strands 1–4,-helix1,-strands 5–8,-helix2). Strands 1 and 8 are held together through a network of hydrogen bonds, giving the protein an overall structure of an asymmetric β-barrel (95). From the structure of OspE complex with FH CCPs 19 to 20, it is clear that the OspE-binding site on FH overlaps with the binding site of heparin, mimicking the natural ligand. Residues making up the core of the OspE interaction are present on β-strands 2 to 4 and are D73, R66, N77, Y114, and S82 (Fig. 3). CCPs 19 to 20 on FH bind to heparin on endothelial cells. The OspE-binding site on FH overlaps with the natural heparin-binding site, indicating that *B. burgdorferi* mimics the host cells in order to bind FH. This is similar to *N. meningitidis* binding CCP 6 to 7. These sites do not change because they are functionally critical. Binding of OspE to its binding site not only leaves the functional CCPs 1 to 4 untouched but also leaves the C3b-binding site available. This is exemplified by a recent example of the tripartite structure (96).

### The Vn-binding SLP, protein E from *H. influenzae*

*H. influenzae* is an important respiratory pathogen that can cause meningitis and sepsis (97). Like *N. meningitidis*, *H. influenzae* is divided into serotypes that are either encapsulated or noncapsulated. Current vaccine formulations do not provide protection for the noncapsulated serotypes of *H. influenzae*, and an SLP-based vaccine may be more effective in combating these strains (98). Our recent bioinformatics search for Slams predicted *H. influenzae* to have a Slam in the proximity of the SLPs (36), including Protein H, an antigen present in recent vaccine formulations undergoing phase two clinical trials (99). *H. influenzae* also displays Protein E, a 16-kDa SLP, responsible for binding several host factors including the complement regulator Vn (100). Vn is a multifunctional 75-kDa glycoprotein that functions to regulate a later stage of complement than FH. Similar to FH, Vn binds directly to complement proteins to modulate their function. Vn is proposed to bind the C5b-7 complex, preventing its insertion into the target membrane, and inhibit the attachment and polymerization of C9 (101). Surface plasmon resonance detected that recombinant Protein E was able to bind immobilized Vn with a k_d_ of 400 nM (102). A lipid anchor–free protein E exists in solution as a dimer, a stoichiometry that was maintained in the asymmetric unit of the crystal structure (103). The protein E monomer is made up of a long alpha helix that is packed into the concave face of a β-sheet comprised of 6 antiparallel β-strands (Fig. 3). The alpha helix is associated to the β-sheet through electrostatic interactions but is also tethered to the sheet through a conserved disulphide bridge (residues 99–148). The protein E dimer in the asymmetric unit of the crystal packs together on the convex side of the β-sheet through a surface that only comprises 625 Å^2^.

There is no current structure of Protein E in complex with Vn, though the core of protein E (84–108) was found to be the site of Vn binding. K85 and R86 of Protein E were shown to be critical for interaction with Vn (104).

## Discussion

The widespread emergence of antibiotic resistance in bacterial pathogens necessitates the need for novel therapeutics and preventative measures (105). Recent advances in the field of bacterial SLP biology has discovered that pathogens display these proteins on their surface where they carry out fundamental roles in bacterial survival, such as overcoming host nutritional immunity and the complement system. SLPs represent a class of underutilized and attractive candidates for therapeutic development, as they are constitutively surface exposed. Indeed, there have been several examples of lipoproteins being evaluated in vaccines (106, 107, 108, 109).

SLPs are likely going to be targets better suited for vaccine development rather than more traditional small-molecule inhibitors. The design of small molecule–based inhibitors targeting SLPs involved in nutrient acquisition would be difficult because a significant amount of sequence variation may preclude the binding of a broad spectrum inhibitor. Efficacy of a small molecule may also be short-lived owing to development of resistance. Furthermore, redundancy is present in SLPs that bind complement regulatory proteins. For example, many *Borrelia* SLPs are geared toward complement evasion (110). Ergo designing a single therapeutic to inhibit all expressed proteins would be challenging. Additionally, therapeutics designed to mimic ligands of SLPs could be potentially hazardous as SLPs bind ligands at positions that are used in physiologically relevant interactions. As demonstrated in the examples discussed above, FHbp, protein E, OspE, and TbpB all bind to their respective ligands at positions that are also used by the host (46, 87, 95, 100). Despite these concerns, recent work using phage display has discovered peptide-based inhibitors of the *N. gonorrheae* SLP AniA, demonstrating that small-molecule inhibitors are still a possibility for some SLPs (111).

**Figure 4:**
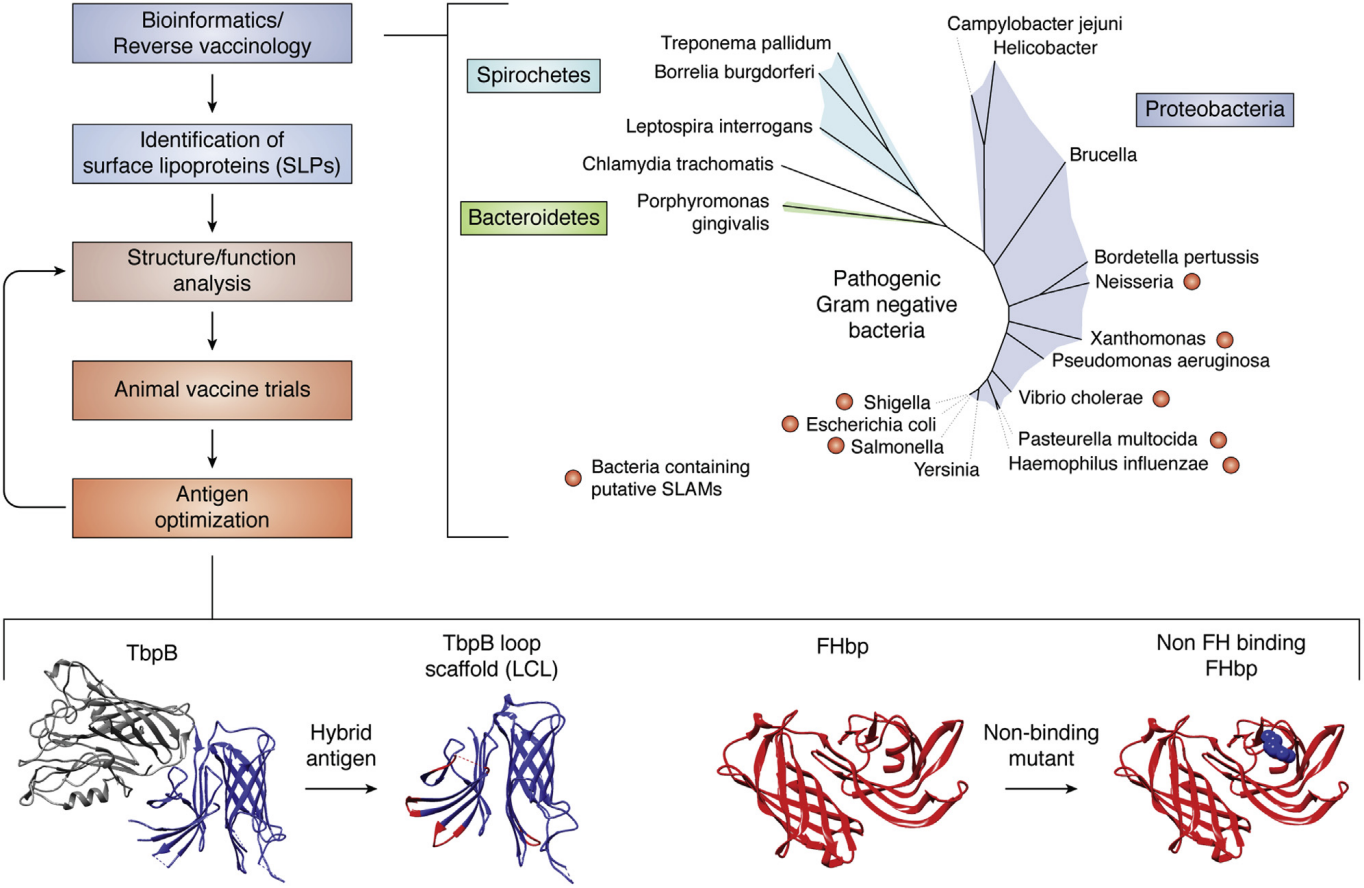
**Schematic diagram of SLP vaccine development pipeline.** Bioinformatics and reverse vaccinology can be used to identify candidate SLPs. This can be facilitated by searching for translocation machinery such as Slams. Pictured is a pruned phylogenetic tree (see text for details) of pathogenic gram-negative bacteria. Bacteria containing putative Slams are indicated with *red dots*. Notice that Slams have not been discovered in the more phylogenetically distant Spirochetes and Bacteroidetes, implying novel translocation machinery may be present in these bacteria. Once a candidate SLP is chosen, structure–function analysis can be used to generate optimized antigens. Two examples include the TbpB-based scaffold, LCL, which has been used to generate TbpA–TbpB hybrids (118), as well asnon-binding FHbp. Both of these examples have improved antigenic properties. This antigenic optimization process can be repeated iteratively to create superior antigens acceptable for human vaccine trials. LCL, loop-less C-lobe; SLP, surface lipoprotein.

Like all vaccines, the development of an SLP-based vaccine is a long and difficult process, as outlined in Figure 4. Important first steps include finding new antigens, a goal that can be obtained through bioinformatics and reverse vaccinology, both powerful tools for the discovery of new antigens (112). Searching genomes for the characteristic lipobox at the N terminus of SLPs can be useful in finding novel lipoproteins but says little about their subcellular localization, which must be experimentally validated. Searching for OM translocation machineries (*i.e.*, Slams) in the genomic proximity of SLPs has been used as a strategy to discover novel SLPs (36). This strategy could be applied to a wide variety of pathogenic bacteria where putative Slams have been discovered. Shown in Figure 4 is a pruned global phylogenetic tree based on a concatenated alignment of 31 universal genes (113). Species containing a putative Slam may produce novel SLPs and are indicated on the tree by a red dot (36). However, this method is limited to Slam homologs and will overlook SLPs that are translocated by unknown methods. For example, Spirochetes and *Bacteroides* have both been hypothesized to have a Slam-like ‘flippase’ in the OM, suggesting a novel translocation system has yet to be discovered (114, 115). Bioinformatics can also reveal the sequence variance of a target within the population of circulating strains. For instance, FHbp can be divided into 2 subfamilies which have >83% sequence identity within each subfamily but only 65 to 73% identity between subfamilies (116). Care must be taken to ensure a vaccine formulation is representative of circulating strains. In the case of a novel SLP discovery, researchers can use bioinformatics to choose a central variant that may result in cross protection across all strains. Alternatively, this information can be used to select how many variants will be required to increase strain coverage.

Once a candidate SLP sequence is chosen, exploring its structure–function relationships is an important next step. Structural elucidation of SLP–ligand complexes has proven to be a very useful tool in solving mechanisms and designing nonbinding mutants, which have enhanced immunogenic properties (91, 117, 118). Although the number of solved SLP structures continues to grow (Table 1), a large number of SLPs remain to be structurally characterized. SLP–ligand complex structures can be difficult to solve, especially for SLPs that bind complex glycoproteins such as FH and Vn. These host ligands are large and heavily glycosylated proteins that are not amenable to crystallography. There are, however, several reports that use a divide and conquer approach to successfully address these difficulties, by isolating only the interacting regions of the host-binding partners and cocrystallising them with their SLP-binding partner (58, 87, 95). The ‘resolution revolution’ in cryo-electron microscopy could provide an alternative pathway to SLP–ligand complex structures, especially as the resolution of samples below 100 kDa improves (119).

**Table 1:** Overview of bacterial SLPs of known structure

Organism	SLP	Function	PDB	Reference
*Actinobacilus pleuropneumoniae*	TbpB	Nutrient acquisition	3HOL	(121)
*Bacteroides thetaiolaomicron*	BT2657	Pilus formation	4QDG	(122)
	BT2263	Pilus formation	5FQ4	(122)
	SusD	Nutrient acquisition	3CKC	(72)
*Borrelia burgdorferi*	BB0365	Unknown	6RIG	(123)
	BB0689	Unknown	4D53	(124)s
	BBA15 (OspA)	Cell survival in ticks	2G8C	(125)
	BBA16 (OspB)	Cell survival in ticks	1RJL	(126)
	BBA24 (DbpA)	Heparin binding	4ONR	(127)
	BBA65	Unknown	4BG5	(128)
	BBA66	Unknown	2YN7	(129)
	BBA68 (Crasp-1/CspA)	Complement evasion	4BL4	(130)
	BBA69	Unknown	6QO1	(131)
	BBA73	Unknown	4AXZ	(132)
	BBB19 (OspC)	Cell migration	1G5Z	(133)
	BBE31	Unknown	6FXE, 6FZE	(134)
	BBH06 (Crasp-2, CspZ)	Complement evasion	4BGO, 4CBE	(135)
	BBK32	Complement evasion	6N1L	(136)
	BBN38 (Crasp-3, ErpP, OspE)	Complement evasion	4J38	(137)
	ErpC (Crasp-4)	Complement evasion	4BXM, 4BOD, 4BF3	(137)
	VlsE	Unknown	1L8W	(138)
*Borrelia turicatae*	VspA/Vsp1	Unknown	1YGJ	(139)
*Borrelia spielmani*	BSA64	Unknown	6HPN	(128)
*Campylobacter jejuni*	JlpA	Cell adhesion	3UAU	(140)
	Cj0090	Unknown	4GIO	(141)
*Escherichia coli*	LptE	LPS assembly	4NHR	(142)
*Francisella tularensis*	Flpp3	Unknown	6PNY	(143)
*Haemophilus parasuis*	TbpB	Nutrient acquisition	4O4X	(117)
*Haemophilus influenza*	Protein E	Complement evasion	6GUS	(103)
*Helocobacter pylori*	Lpp20	Unknown	5OK8	(144)
*Kingella denitrificans*	HpuA	Nutrient acquisition	5EE4	(67)
*Klebsiella pneumoniae*	PulA	Nutrient acquisition	2YOC	(28)
	LptE	LPS assembly	5IV9	(145)
*Legionella pneumophila*	MIP	Proline isomerase	1FD9	(146)
*Leptospira interrogans*	LipL32	Cell adhesion	2ZZ8	(147)
*Leptospira santarosai*	LipL32	Cell adhesion	2WFK	(148)
*Yersinia pestis*	LptE	LPS assembly	5IXM	(145)
*Neisseria gonnorehae*	AniA	Anaerobic respiration	5UE6	(111)
*Neisseria meningitidis*	AniA	Anaerobic respiration	1KBW	(149)
	FHBP	Complement evasion	2w80	(87)
	FrpD	Unknown	5EDJ	(150)
	GNA 1162	Unknown	4HRV	(151)
	NHBA	Complement evasion	6CUJ	(81)
	TbpB	Nutrient acquisition	3PQU	(46)
	LbpB	Nutrient acquisition	1lfg	(58)
*Porphromonas gingivatis*	RagB	Cell survival	5cx8	(152)
	Ihtb	Nutrient acquisition	5Y1A	(153)
*Pseudomonas aeruginosa*	LptE	LPS assembly	2N8X	(145)
*Treponema pallidum*	TP0435	Unknown	4U3Q	(154)
*Xanthomonas citri*	OmlA	Unknown	2PXG	(155)

SLP, surface lipoprotein.

With an SLP-based antigen in hand, an initial animal vaccine trial can be done to assess safety, immunogenicity, and dosage. This can be done in parallel with further structural studies that could give insights into what possible mutations/alterations should be made to the antigen. The generation of nonbinding mutants, such as FHbp R106S (Fig. 4), can result in antigens with superior immunogenic properties (91). Mutations can also be used to optimize the fold stability of the antigen, which can have a profound effect on the amount of protection a vaccine can produce (120). The design and construction of hybrid antigens can also improve immunogenicity. Our group has shown that the TonB-dependent receptor TbpA of *N. meningitidis* is more well conserved across all strains relative to its SLP counterpart, TbpB. This information was used to engineer a novel antigen in which four loops on the C-lobe of TbpB were shortened to create a scaffold (the loop-less C-lobe or LCL) (Fig. 4) onto which the more conserved TbpA loops were grafted (118). In the case of some loops, a more cross-protective antigen was created. The above-mentioned processes can be carried out in an iterative manner (Fig. 4), with gradual improvements, until a final vaccine formulation is reached and is ready for human trials.

## Conflict of interest

T. F. M. is a coinventor on a patent that describes the design and production of hybrid antigens and is a cofounder of Engineered Antigens Inc. The remaining authors declare no conflict of interest.
